# Long-term trends of visceral leishmaniasis incidence and mortality in India 1990–2019: an application of joinpoint and age-period-cohort analysis

**DOI:** 10.1186/s12879-025-10751-7

**Published:** 2025-09-29

**Authors:** Deepak Dhamnetiya, Krittika Bhattacharyya, Ravi Prakash Jha, Neha Shri, Mayank Singh, Priyanka Patel

**Affiliations:** 1 Department of Community Medicine, Atal Bihari Vajpayee Institute of Medical Sciences (ABVIMS)and, RML Hospital (RML), New Delhi, 110001 India; 2 Department of Statistics, University of Calcutta, Kolkata, 700019, India; 3 Department of Community Medicine, Dr. Baba Saheb Ambedkar Medical College & Hospital, Delhi, India; 4 International Institute for Population Sciences (IIPS), Mumbai, 400088, India; 5 Department of Epidemiology and Biostatistics, KLE Univeristy, Belagavi, 590010 India; 6 Newcomb Institute Tulane University, New Orleans, LA USA

**Keywords:** Visceral Leishmaniasis, Incidence, Mortality, Age-Period-Cohort, India

## Abstract

Leishmaniasis, a vector-borne disease, remains one of the most significant parasitic disease with potential outbreak and high mortality. Despite improvements in living standards and health infrastructure, there has been a territorial expansion in the incidence and lethality of the disease. However, evidence regarding its control, prevention, and eradication remains limited. Given an understanding of the present status of the disease, this study assesses the trends and patterns in the incidence and mortality of this endemic in India from 1990 to 2019. This study obtained Visceral Leishmaniasis (VL) incidence and mortality data (1990–2019) from the Global Burden of Disease (GBD) study 2019, provided by the Institute for Health Metrics and Evaluation (IHME). To capture the overall changes and sex-specific changes in ASIRs and ASMRs of VL, joinpoint regression analysis was employed for all ages by using joinpoint regression programme version 4.5.0.1. Age-period-cohort analysis is used to estimate the net age, period, and cohort effects on the incidence and mortality of VL from observed age-specific incidence and mortality rates. The APC model was implemented using Stata 16.0, and its fit was assessed using Akaike’s Information Criterion (AIC) and Bayesian Information Criterion (BIC). Findings indicate a significant decline in the age-specific incidence and mortality rates for both sex. The highest annual percentage decline in VL incidence and mortality was observed during 2011–2016 for both males and females. (Table 1). Results from the age effect show that the risk of VL incidence and mortality among both genders decreased sharply with advancing age. Period effect indicated a sharp decline in incidence and mortality risk from the period 1990–94 to 2000–04. The cohorts effect showed that compared to earlier birth cohort (1900–04),, the relative risk (RR) of VL incidence increased by 1.5 in male and 1.7 times in females in more recent birth cohort (2015–19), whereas mortality risk decreased by 48% among females. Findings highlight a notable reduction in VL incidence and mortality in the country. The age pattern in the incidence and mortality rates indicates the need for age-specific attention while performing preventive measures and comprehensive strategies. Moreover, strengthening large-scale screening, vector control measures, and public health education is essential to sustain VL elimination efforts.

**Supplementary Information:**

The online version contains supplementary material available at 10.1186/s12879-025-10751-7.

## Introduction

Leishmaniasis vector-borne disease is one of the ten neglected tropical diseases globally and is considered a disease of developing nations that contributes to mortality and morbidity significantly [34, 40]. Visceral leishmaniasis (VL) or named Kala-azar in the Indian Sub-continent, is a lethal form of Leishmaniasis disease is caused by the protozoan parasite *Leishmania Donovani* and is transmitted from one person to another person by the bite of infected female sandfly known as *Phlebotomus aregentipes*, [6, 27, 41]. Another mode of transmission of infections was mother to fetus and the use of infected needles which is often the case with the HIV-VL co-infection. In 2019, more than 90% of the cases that occurred worldwide were concentrated in ten countries, and India is one of them WHO [22] and remains one of the major eco-epidemiological hotspots of the world. Characterized by irregular bouts of fever, weight loss, anemia and enlarged spleen and liver, disability and death caused by the disease can be prevented by complete and early treatment. The severity of the endemic can be understood by the statement that this disease if untreated has a mortality rate of 75–95% [16, 19]. Furthermore, the fatalities vary according to factors such as age, gender, and residence [18, 26]. According to WHO, the epidemiology of this disease is influenced by social, environmental, and climatologic factors and poverty. Although the incidence of visceral leishmaniasis has been declining in areas where living standards have improved, this endemic continues to kill thousands of people infected with *Leishmania Donovani*on in the Indian subcontinent and East Africa. Many countries have experienced an increase in the incidence and lethality with a territorial expansion of this endemic [8]

India witnessed numerous outbreaks of kala-azar in the country in the nineteenth century [42]. As per the estimates published by WHO in 2017, the incidence rate of this endemic was 0.62 cases per 10,000 population, and around 10% of the total population was at risk of developing the disease in the year 2015 [48]. The guidelines issued by the National Vector Borne Disease Control Programme state that 5758 cases of kala-azar were reported in 2017 and no deaths have been reported in the country since 2018 [30]. According to Weekly Epidemiological Record, in 2020, there were 2048 cases of kala-azar reported in the country. Additionally, the case fatality rate was found to be 1.3% in 2019 and 1.8% in 2020. Further, around 57–60% of the kala-azar cases were reported among males and around 40% of the cases were among socially disadvantaged classes. Since, this disease mostly occurs among socially less privileged communities residing on the outskirts of villages [37], there is no doubt why this disease has been termed as a rural poor man’s disease in India [38]. The disease is primarily prevalent in Bihar, West Bengal, Uttar Pradesh, Madhya Pradesh, Jharkhand, Kerala, and Gujarat, with Bihar accounting for nearly half of the total cases in the Indian subcontinent. Bihar only accounted for approximately half of the overall cases across the Indian subcontinent [20]. However, in recent years, the epidemiology of this disease has spread in western states such as Uttarakhand and Himachal Pradesh. Studies have reported that despite being infected with the disease, most people do not develop the disease and remain asymptomatic [39]. Research suggests that young children and older adults are at higher risk of getting infected [2]. According to recent estimates, roughly 165.4 million people in Bihar, Uttar Pradesh, Jharkhand, and West Bengal are at risk of contracting kala-azar [30].

Concerned with the increasing incidence of Kala-azar in the country, the government of India undertook the Kala-azar elimination program under the National Health Mission −2002 with the target of reducing the annual incidence to less than one per 100,000 population at the block level by 2015, the primary goal is supposed to be achieved by the year 2010 [31]. The target India has achieved the target in more than 70% of the endemic district by the year 2015 [48]. Despite the considerable work in the direction of elimination of VL disease several programmes have failed [3, 30, 42]. The prevalence of this disease and the regions of endemic has increased in recent years [11]. For instance, the incidence of kala-azar peaked in 1987 & 2004 in West Bengal [1, 5]. Goal 3 of SDG aims to end the epidemic of neglected tropical diseases by 2030 [43].

This disease continues to be an obstacle to the development of the country from a socio-economic perspective. Since, India lacks comprehensive data on this disease, further step towards the control, prevention, and eradication of the endemic requires scrutiny of its incidence and mortality. Nonetheless, the information available on the incidence and mortality of the disease in India is scanty. In view of understanding the present status of the disease, this study assesses the trends and patterns in the incidence and mortality of this endemic in India from 1990 to 2019.

## Materials and methods

### Data source

This study obtained Visceral Leishmaniasis incidence and mortality data (1990–2019) from the global burden of disease (GBD) study 2019, which was provided by the Institute for Health Metrics and Evaluation (IHME, http://www.healthdata.org/). The GBD 2019 provides comprehensive estimates of annual incidence, prevalence, mortality, YLLs, YLDs, and DALYs for 369 diseases and injuries, which are reported by sex, location, and age group for 204 countries and territories that are grouped in 21 regions and 7 super-regions. The Cause of Death Ensemble model (CODEm) and spatial–temporal Gaussian process regression is used to generate cause-specific death rates for a variety of causes and cause fractions. A detailed description of CODEm is reported here [13, 24, 28, 29]. The GBD study's goal is to create a set of global health measurements that are both comprehensive and comparable. For Visceral Leishmaniasis in India, 59 distinct data sources were utilized to model the cause of death, and 62 different data sources were used to model both causes of death and disability estimates. Medical certification of the cause of death of the country and of various states, WHO Global Health Observatory reported cases, vital statistics reports, other surveys on the cause of death, and published scientific studies were the primary data sources used to model the cause of death related to Visceral Leishmaniasis in India [12, 44]. In our study, we exclusively discuss a specific type of Leishmaniasis called Visceral Leishmaniasis. The incidence rate and mortality rate of Visceral Leishmaniasis were taken from the GHDx (Global Health Data Exchange) query tool, which was created by the IHME and is publicly available at (http://ghdx.healthdata.org/gbd-results-tool) [15]**.** This study uses GBD 2019 data, which may have biases due to reporting differences, underreporting in rural areas, and variations in diagnostic accuracy. Model-based estimates introduce uncertainties, especially in regions with limited data. Limited primary data collection in marginalized communities may underestimate the VL burden. Ethically, equitable healthcare access is crucial. Disparities in disease reporting, diagnosis, and treatment access necessitate improved surveillance and stronger healthcare systems.

### Statistical analysis

#### Joinpoint regression analysis

Joinpoint regression analysis was employed to evaluate temporal trends in VL incidence and mortality, identifying significant inflection points. This method helps identify specific time points (joinpoints) where trends significantly change, rather than assuming a steady increase or decrease over time. Unlike traditional piecewise or segmented regression models, which require pre-specified breakpoints, joinpoints in this method are determined within the model itself.

This analysis identifies segments within the time series where statistically significant changes occur. For each segment, the model calculates the average percentage change (APC), which quantifies the rate of change between two joinpoints. If no joinpoints are found, the Average Annual Percentage Change (AAPC) is used to summarize the overall trend. The model is formulated as:

$$\text{log}\left({\text{Y}}_{\text{x}}\right)={\text{b}}_{0}+{\text{b}}_{1\mathbf{X},}$$

where log (Y_x_) is the natural logarithm of the rate in year x. Then the APC between year x to x + 1 is defined as

$$APC=\frac{{e}^{{b}_{0}+{b}_{1}(x+1)}-{e}^{{b}_{0}+{b}_{1}x}}{{e}^{{b}_{0}+{b}_{1}x}}*100= \left({e}^{{b}_{1}}-1\right)*100$$

If no joinpoints are identified, the APC remains constant, and this constant value is referred to as the average annual percentage change (AAPC). When joinpoints are present, the time series is divided into segments at these points. The AAPC is then calculated as a geometric weighted average of the APCs for each segment, with the weights based on the lengths of the segments, following the method described by Clegg et al. [10].

The statistical significance of the APC and AAPC is tested using the Z test. The terms 'increase', 'decrease', or 'stable' are used to describe trends, depending on whether the slope (APC or AAPC) is statistically significant or not. The analysis was conducted using the Joinpoint Regression Program (version 4.5.0.1) from the U.S. National Cancer Institute Kim et al. [21], allowing up to five joinpoints to comprehensively capture trend changes. Age Period Cohort Analysis.

The Age Period Cohort (APC) analysis is a robust statistical method employed to dissect the layered effects of age, period, and cohort on health outcomes, notably the incidence and mortality rates of Visceral Leishmaniasis (VL) over time. When assessing the incidence and mortality associated with Visceral Leishmaniasis, typical statistical analyses are unable to break down these cumulative risks and health hazard, as highlighted in studies by Luo et al. [25] and Wang et al. [46]. This technique is crucial in differentiating the risks associated with VL that accumulate over time due to various social, environmental, and historical influences on individuals and population groups [51].

APC analysis elucidates the distinct yet intertwined impacts of an individual's age, the time period, and their birth cohort [45, 47, 50], Wang et al. [46]; [23]. Given the inherent challenge known as the identification problem—where the three variables are linearly dependent (cohort equals period minus age)—the Intrinsic Estimator (IE) method is utilized [14, 50]. This approach helps to reliably tease apart these intertwined effects, providing an unbiased and efficient estimation of the temporal trends affecting VL incidence and mortality rates.

The APC model categorizes age-specific incidence and mortality data into sequential five-year age groups (ranging from 0–4 to 90–94 years), five-year periods (1990–1994 to 2015–2019), and corresponding five-year birth cohorts (1900–1904 to 2015–2019). The objective is to accurately estimate the net effects of age, period, and cohort on the incidence and mortality of VL.

The mathematical representation of the APC model is given by:

$$\text{Y}=\text{log}\left(\text{M}\right)=\upmu +{{\alpha age}}_{\text{i}}+{{\beta period}}_{\text{j}}+{{\gamma cohort}}_{\text{k}}+\upvarepsilon$$

Here, M denotes the incidence and mortality for each age group, µ is the model's intercept α, β, and γ are the coefficients representing age, period, and cohort effects respectively, and Ɛ denotes the residual of the APC model.

To implement the APC analysis, Stata 16.0 software is used, with model fit assessed through metrics such as deviance, Akaike's Information Criterion (AIC), and the Bayesian Information Criterion (BIC). These helps evaluate the model's adequacy in capturing the complex dynamics of VL incidence and mortality. The analysis also involves calculating risk ratios and the standard errors of the coefficients to quantify the influence of each variable.

The APC model was executed using Stata 16.0 software, provided by Stata Corp, based in College Station, TX, USA. To evaluate the model's fit, various statistical measures were applied, including deviance, Akaike’s Information Criterion (AIC), and the Bayesian Information Criterion (BIC). Additionally, risk ratios and the standard errors (SE) of the coefficients were calculated to estimate the precision and reliability of the model outcomes.

## Results

**Fig. 1:**
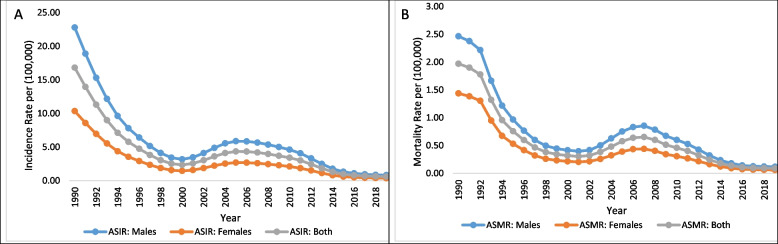
Age-standardized incidence and mortality rates of Visceral Leishmaniasis in India

Trends in age-standardized incidence and mortality of VL by gender at all aggregate ages from 1990 to 2019 are displayed in Fig. 1A and B respectively. Estimates show that ASIR in males was 22.8 per 100,000 during 1990 and it reduced to 0.8 per 100,000 in 2019, similarly, among females, ASIR was observed to be 10.4 per 100,000 in 1990 and reached 0.4 per 100,000 in 2019 (Fig. 1A). The age-standardized mortality rate of VL decreased from 2.4 and 1.4 per 100,000 in 1990 to 0.4 and 0.2 per 100,000 in 2001 among males and females respectively. Subsequently, the mortality rates increased after the year 2001 and reaches 0.85 and 0.44 per 100,000 in 2007 for males and females respectively, thereafter again started declining achieving the minimum of 0.12 and 0.06 per 100,000 in 2019 among males and females respectively (Fig. 1B).

**Fig. 2:**
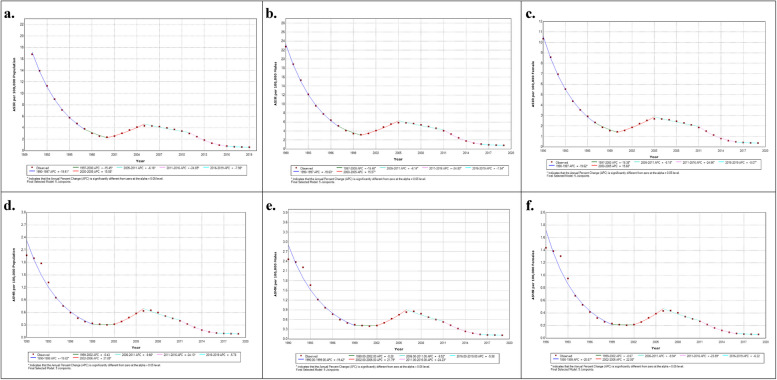
Sex-specific temporal trends in age-standardized incidence and mortality of Visceral Leishmaniasis in India based on the joinpoint regression analysis (1990–2019). Note: **a**. incidence of both sexes, **b**. Incidence male, **c**. incidence female, **d**. mortality both sexes **e**. mortality male, **f**. mortality female

Table 1 and Fig. 2 illustrate the trends in incidence and mortality of VL in India by gender, as obtained by the join points analysis. Row named AAPC indicates the trends in the whole period 1990–2019. Both incidence and mortality of VL decreased significantly with an APPC of −10.96 (95% CI: −11.58, −10.35) and −10.69 (95% CI: −13.59, −7.69) respectively. The highest annual percentage decrement in VL incidence and mortality was found in the period 2011–16 with an APC of −24.80 (95% CI: −26.0, −23.58) in males and −24.96 (95% CI: −26.16, −23.74) in females for incidence and for mortality −24.23* (95% CI: −29.4, −18.7) in males and −23.89* (95% CI: −29.59, −17.73) in females. An increasing trend in VL was also observed during the period 2000–05 and 2002–06 in case of incidence and mortality respectively.

**Table 1:** Trends in Visceral Leishmaniasis incidence and mortality in India from 1990 to 2019 using joinpoint regression analysis

ASIR per 100,000 Population			ASIR per 100,000 Males			ASIR per 100,000 Females		
Segment	Year	APC* (95% C.I.)	Segment	Year	APC* (95% C.I.)	Segment	Year	APC* (95% C.I.)
1	1990–1997	-19.61* (-20.16, -19.07)	1	1990–1997	-19.63* (-20.18, -19.09)	1	1990–1997	-19.62* (-20.17, -19.07)
2	1997–2000	-15.45* (-19.64, -11.05)	2	1997–2000	-15.46* (-19.64, -11.07)	2	1997–2000	-15.38* (-19.59, -10.96)
3	2000–2005	15.58* (13.74, 17.45)	3	2000–2005	15.57* (13.73, 17.44)	3	2000–2005	15.68* (13.83, 17.56)
4	2005–2011	-6.16* (-7.22, -5.09)	4	2005–2011	-6.14* (-7.19, -5.07)	4	2005–2011	-6.18* (-7.24, -5.1)
5	2011–2016	-24.85* (-26.05, -23.64)	5	2011–2016	-24.80* (-26, -23.58)	5	2011–2016	-24.96* (-26.16, -23.74)
6	2016–2019	-7.98* (-10.28, -5.61)	6	2016–2019	-7.94* (-10.24, -5.58)	6	2016–2019	-8.07* (-10.38, -5.69)
AAPC*	1990–2019	-10.96* (-11.58, -10.35)	AAPC*	1990–2019	-10.95* (-11.56, -10.33)	AAPC*	1990–2019	-10.98* (-11.59, -10.36)
ASMR per 100,000 Population			**ASMR per 100,000 Males**			**ASMR per 100,000 Females**		
Segment	Year	APC* (95% C.I.)	Segment	Year	APC* (95% C.I.)	Segment	Year	APC* (95% C.I.)
1	1990–1999	-19.82* (-21.49, -18.11)	1	1990–1999	-19.42* (-21.04, -17.76)	1	1990–1999	-20.57* (-22.33, -18.76)
2	1999–2002	-0.43 (-20.97, 25.45)	2	1999–2002	-0.28 (-20.22, 24.65)	2	1999–2002	-0.67 (-22.34, 27.04)
3	2002–2006	21.85* (8.56, 36.77)	3	2002–2006	21.79* (8.94, 36.17)	3	2002–2006	22.08* (7.94, 38.06)
4	2006–2011	-9.66* (-16.02, -2.81)	4	2006–2011	-9.52* (-15.69, -2.91)	4	2006–2011	-9.94* (-16.68, -2.65)
5	2011–2016	-24.13* (-29.47, -18.38)	5	2011–2016	-24.23* (-29.4, -18.7)	5	2011–2016	-23.89* (-29.59, -17.73)
6	2016–2019	-5.78 (-16.06, 5.75)	6	2016–2019	-5.58 (-15.55, 5.56)	6	2016–2019	-6.22 (-17.07, 6.06)
AAPC*	1990–2019	-10.69* (-13.59, -7.69)	AAPC*	1990–2019	-10.52* (-13.33, -7.62)	AAPC*	1990–2019	-10.99* (-14.07, -7.8)

Note: *, Indicates that the Annual Percent Change (APC) is significantly different from zero at the alpha = 0.05 level

**Fig. 3:**
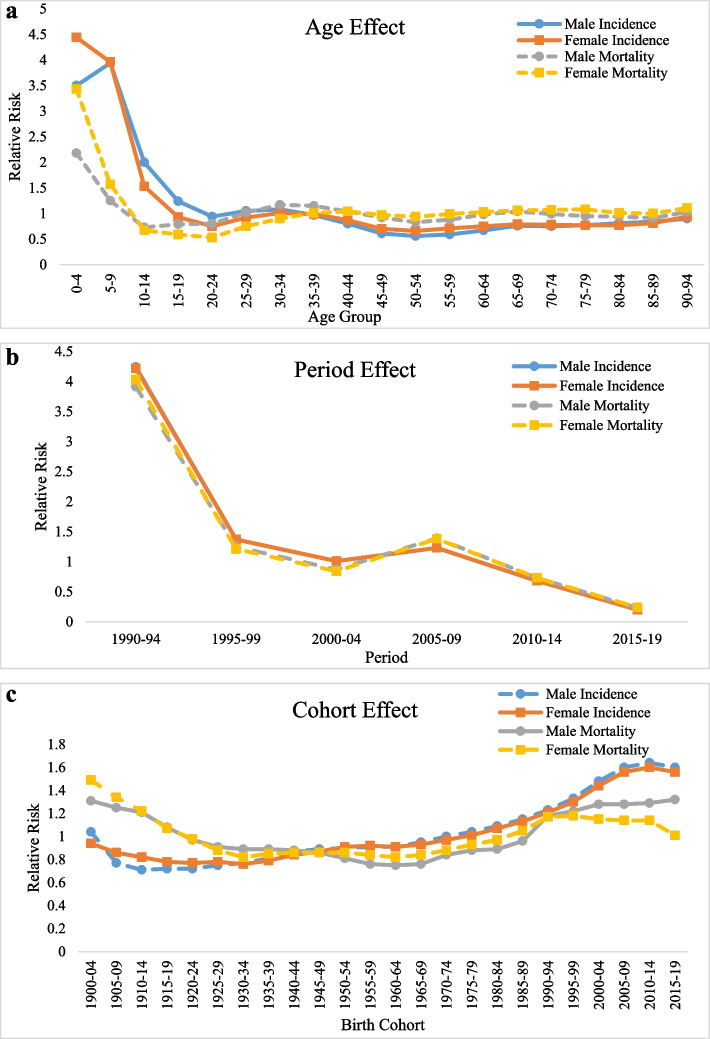
The incidence and mortality relative risks of Visceral Leishmaniasis due to (**a**) age; (**b**) period; and (**c**) cohort effects

The relative contribution of age, period and birth cohort effect on VL incidence and mortality in India is displayed in Fig. 3, Table 2. The APC-IE analysis estimated coefficients for the age, period, and cohort effects (Appendix Table 1). Further, these coefficients were then calculated to their exponential value (exp(coef.) = ecoef.) that denotes the incidence and mortality relative risk (RR) of a particular age, period, or birth cohort relative to each average level (Table 2).

**Table 2:** The incidence and mortality relative risks of Visceral Leishmaniasis due to age, period, and cohort effects

Factors	Incidence (RR 95% CI)		Mortality (RR 95% CI)	
	**Male**	**Female**	**Male**	**Female**
**Age**				
** 0–4**	3.5 [3.37 3.63]	4.44 [4.24 4.64]	2.18 [1.92 2.47]	3.43 [3.23 3.64]
** 5–9**	3.94 [3.82 4.07]	3.96 [3.81 4.12]	1.25 [1.12 1.4]	1.57 [1.49 1.65]
** 10–14**	2 [1.94 2.05]	1.53 [1.48 1.59]	0.73 [0.66 0.8]	0.67 [0.64 0.7]
** 15–19**	1.24 [1.21 1.27]	0.93 [0.9 0.96]	0.79 [0.72 0.86]	0.59 [0.56 0.61]
** 20–24**	0.94 [0.92 0.96]	0.75 [0.73 0.77]	0.8 [0.74 0.87]	0.53 [0.51 0.56]
** 25–29**	1.05 [1.03 1.07]	0.92 [0.89 0.94]	1.02 [0.95 1.11]	0.75 [0.72 0.78]
** 30–34**	1.08 [1.06 1.1]	1.01 [0.99 1.04]	1.17 [1.08 1.26]	0.9 [0.86 0.93]
** 35–39**	0.97 [0.95 0.99]	0.99 [0.96 1.01]	1.15 [1.05 1.25]	1.01 [0.97 1.05]
** 40–44**	0.8 [0.78 0.82]	0.87 [0.84 0.89]	1.04 [0.95 1.14]	1.04 [0.99 1.08]
** 45–49**	0.61 [0.59 0.63]	0.7 [0.67 0.72]	0.92 [0.83 1.02]	0.97 [0.92 1.02]
** 50–54**	0.56 [0.54 0.58]	0.66 [0.63 0.69]	0.83 [0.73 0.94]	0.94 [0.88 0.99]
** 55–59**	0.59 [0.57 0.62]	0.71 [0.67 0.74]	0.88 [0.77 1.01]	0.99 [0.93 1.05]
** 60–64**	0.67 [0.64 0.7]	0.75 [0.71 0.79]	0.98 [0.84 1.13]	1.03 [0.96 1.1]
** 65–69**	0.76 [0.72 0.8]	0.79 [0.75 0.84]	1.04 [0.88 1.22]	1.06 [0.98 1.14]
** 70–74**	0.75 [0.71 0.8]	0.78 [0.73 0.83]	0.99 [0.82 1.19]	1.07 [0.98 1.17]
** 75–79**	0.77 [0.71 0.82]	0.77 [0.71 0.83]	0.95 [0.76 1.19]	1.08 [0.97 1.19]
** 80–84**	0.81 [0.74 0.89]	0.77 [0.69 0.85]	0.94 [0.7 1.25]	1.01 [0.88 1.15]
** 85–89**	0.84 [0.73 0.96]	0.81 [0.7 0.94]	0.91 [0.59 1.42]	1 [0.82 1.22]
** 90–94**	0.9 [0.68 1.19]	0.94 [0.7 1.27]	1.03 [0.44 2.46]	1.11 [0.75 1.65]
**Period**				
** 1990–94**	4.24 [4.18 4.31]	4.22 [4.15 4.3]	3.91 [3.71 4.11]	4.03 [3.94 4.13]
** 1995–99**	1.37 [1.35 1.38]	1.37 [1.35 1.38]	1.25 [1.2 1.3]	1.21 [1.19 1.24]
** 2000–04**	1.01 [1.01 1.02]	1.01 [1 1.02]	0.87 [0.84 0.91]	0.84 [0.82 0.86]
** 2005–09**	1.23 [1.22 1.24]	1.23 [1.22 1.24]	1.39 [1.35 1.44]	1.38 [1.36 1.4]
** 2010–14**	0.68 [0.67 0.69]	0.68 [0.67 0.69]	0.73 [0.7 0.77]	0.73 [0.71 0.74]
** 2015–19**	0.2 [0.2 0.21]	0.2 [0.2 0.21]	0.23 [0.21 0.25]	0.24 [0.23 0.25]
**Cohort**				
** 1900–04**	1.04 [0.64 1.67]	0.94 [0.53 1.68]	1.31 [0.26 6.53]	1.49 [0.71 3.12]
** 1905–09**	0.77 [0.62 0.96]	0.86 [0.67 1.1]	1.25 [0.62 2.53]	1.34 [0.97 1.86]
** 1910–14**	0.71 [0.62 0.81]	0.82 [0.7 0.96]	1.21 [0.79 1.86]	1.22 [1 1.49]
** 1915–19**	0.72 [0.65 0.8]	0.78 [0.7 0.88]	1.08 [0.78 1.51]	1.07 [0.92 1.25]
** 1920–24**	0.72 [0.67 0.79]	0.77 [0.7 0.85]	0.97 [0.74 1.28]	0.98 [0.86 1.12]
** 1925–29**	0.75 [0.7 0.81]	0.78 [0.72 0.85]	0.91 [0.71 1.15]	0.88 [0.78 0.98]
** 1930–34**	0.77 [0.72 0.82]	0.76 [0.71 0.82]	0.89 [0.72 1.11]	0.82 [0.74 0.91]
** 1935–39**	0.81 [0.76 0.86]	0.79 [0.73 0.84]	0.89 [0.73 1.09]	0.85 [0.78 0.94]
** 1940–44**	0.87 [0.82 0.91]	0.84 [0.78 0.89]	0.88 [0.73 1.06]	0.86 [0.79 0.93]
** 1945–49**	0.89 [0.85 0.94]	0.87 [0.82 0.93]	0.86 [0.73 1.01]	0.86 [0.79 0.93]
** 1950–54**	0.9 [0.87 0.94]	0.91 [0.86 0.96]	0.81 [0.7 0.94]	0.86 [0.81 0.93]
** 1955–59**	0.91 [0.87 0.94]	0.92 [0.87 0.96]	0.76 [0.67 0.87]	0.84 [0.79 0.89]
** 1960–64**	0.91 [0.88 0.94]	0.91 [0.87 0.94]	0.75 [0.67 0.84]	0.82 [0.77 0.86]
** 1965–69**	0.95 [0.92 0.97]	0.93 [0.9 0.96]	0.76 [0.69 0.84]	0.84 [0.8 0.88]
** 1970–74**	1 [0.98 1.02]	0.97 [0.94 1]	0.84 [0.77 0.91]	0.88 [0.84 0.91]
** 1975–79**	1.04 [1.03 1.06]	1.01 [0.99 1.04]	0.88 [0.83 0.95]	0.93 [0.9 0.97]
** 1980–84**	1.09 [1.07 1.1]	1.07 [1.05 1.08]	0.89 [0.84 0.94]	0.97 [0.95 1]
** 1985–89**	1.15 [1.14 1.16]	1.13 [1.11 1.14]	0.96 [0.91 1]	1.05 [1.03 1.08]
** 1990–94**	1.23 [1.22 1.24]	1.21 [1.19 1.22]	1.18 [1.13 1.24]	1.17 [1.15 1.2]
** 1995–99**	1.33 [1.31 1.35]	1.3 [1.27 1.32]	1.22 [1.14 1.3]	1.18 [1.15 1.22]
** 2000–04**	1.48 [1.45 1.51]	1.44 [1.41 1.48]	1.28 [1.18 1.38]	1.15 [1.1 1.19]
** 2005–09**	1.6 [1.56 1.64]	1.56 [1.52 1.61]	1.28 [1.16 1.4]	1.14 [1.09 1.19]
** 2010–14**	1.64 [1.58 1.69]	1.6 [1.54 1.66]	1.29 [1.14 1.46]	1.14 [1.08 1.21]
** 2015–19**	1.6 [1.53 1.68]	1.56 [1.48 1.65]	1.32 [1.09 1.59]	1.01 [0.93 1.1]
**Deviance**	**86.19**	**56.02**	**314.19**	**37.75**
**AIC**	**10.80**	**9.72**	**11.27**	**8.11**
**BIC**	**−235.87**	**−266.04**	**−7.87**	**−284.31**

### Age effect

Figure 3a shows the age-adjusted relative risk of VL incidence and mortality. Risk decreases sharply from ages 0–4 to 20–24, then fluctuates slightly. Overall, incidence risk declined 3.9 times in males and 4.7 times in females, while mortality risk dropped 2.1 times in males and 3.1 times in females. Incidence and mortality were generally higher in females (Table 2).

### Period effect

Figure 3b and Table 2 show a significant period effect on VL incidence and mortality. Risk declined sharply from 1990–94 to 2000–04, briefly increased until 2005–09, then decreased again until 2015–19. Over the full period, incidence risk fell 21.2 times in males and 21.1 times in females, while mortality risk declined 17 times in males and 16.8 times in females.

### Cohort effect

Figure 3c and Table 2 show the cohort effect on VL incidence and mortality. In males, mortality risk declined from the 1900–04 to 1960–64 birth cohort, then increased until 2015–19. In females, mortality risk decreased from 1900–04 to 1930–34, then rose until 1995–99, with a later decline. Incidence risk increased steadily from 1910–14 to 2010–14 in males and from 1930–34 to 2010–14 in females. Overall, incidence risk rose 1.5 times in males and 1.7 times in females, while female mortality risk dropped 48%, with no major change in males.

## Discussion

This study was conducted with an aim to analyze the effect of age, period, and cohort on the incidence and mortality caused by visceral leishmaniasis in the country in the period 1990–2019 using the data from the Global Burden of Disease Study. We found that the age-standardized incidence rate and mortality rates of VL have declined substantially among males and females in the period 1990–2000. We found higher age-standardized incidence and mortality rates for males than females. Various studies conducted have observed higher VL incidence in males than females [7, 18, 36] characterized by socio-behavioral and biological factors [7, 36]. We witnessed an increase in the age-standardized incidence and mortality rates of this disease in the period 2000–2008 which declined rapidly onwards. This study findings align with Bhunia and colleagues who also witnessed a rising trend of incidence of this disease during 2005–08, in the country [5]. The government's and health organizations' extensive kala-azar awareness campaigns might have led to increased reporting of kala-azar cases at public health centers (PHCs). However, after the period 2016, the decline was not much pronounced. Further, estimates from AAPC indicated an overall decline in VL incidence and mortality, and a similar pattern was observed for males and females. Studies from India and other developing countries like Nepal and Bangladesh have reported similar declines VL incidence rate in the recent past [49]. However, these studies have also reported a variation in the prevalence rates and incidence determined by geographical location and time period [9]. The long-term trends in the incidence of VL were analyzed using the APC model. An in-depth examination into incidence and mortality relative risks of VL classified by age, period, and cohort indicate varying patterns which have been discussed in subsequent paragraphs.

The varying incidence and mortality rates across different ages, commonly known as the age effect indicate that human age is significantly associated with this disease. The incidence and mortality of visceral leishmaniasis (VL) in India vary significantly by age and gender. Young children (0–9 years) are disproportionately affected, with a notable decline in incidence thereafter—suggesting the development of immunity over time [18, 32]. Although males generally exhibit higher rates in the 10–44-year age group, females show elevated rates in other age brackets, possibly due to differential exposure [4, 33]. Moreover, the lowest mortality rates observed among children aged 10–24 years further support the role of acquired immunity. Similar patterns are seen in other endemic countries like Bangladesh and Nepal, though gender dynamics may vary. In India, VL predominantly affects rural, low-income populations where factors such as malnutrition, population displacement, and inadequate housing compound the disease burden Singh et al. [37]. Consequently, targeted interventions in high-burden regions like Bihar are crucial, especially given the challenges in accessing standard-of-care therapy due to financial and logistical barriers Singh et al. [37].

Period effect: The period trends observed in VL incidence reported here are consistent with previously reported trends in VL. A significant period effect was observed in the incidence and mortality of VL in the country. In the time period 1990–94 to 1995–99, a downward trend was observed in the period RRs of incidence and mortality due to VL. Similar to our findings, a report reported that peak annual incidence was observed in 1992 Disease Control program report [30]. The sharp decline observed in later years coincides with intensified national kala-azar control efforts, including indoor residual spraying and free access to treatment programs. Moreover, the relative risk of incidence was highest in the period 1990–94 while the lowest incidence was observed in the recent period i.e. 2015–19. Lack of comprehensive VL surveillance following the efforts to control the epidemic lead to a resurgence of VL cases in states like Bihar [35]. Similar trends in the period effect were observed for both sexes. State-level studies conducted in the country have reported a significant decline in the number of VL cases since 2011 [18, 30]. Availability and awareness of free diagnosis and treatment of VL might have been effective in reducing the pace of this disease.

The decline in VL incidence has significant implications for healthcare infrastructure and resource allocation. Further, the incidence of the epidemic observed peaks in 1978, 1992, and 2007 which was suppressed largely during the malaria eradication campaigns of the 1950-60 s. The kala-azar control program launched intensified during the year 1990–91 leading to a reduction in the mortality and morbidity in the kala-azar cases. A vigorous campaign of case detection and indoor residual spraying with DDT resulted in this sharp decline in the next two years. The incidence and mortality rates were the same for males and females across all periods. However, studies have reported differences in male and female mortality rates [4, 17].

Cohort effect: In this study, we found evidence supporting the existence of a cohort effect in the VL incidence and mortality caused by VL in India in the period 1990–2019. In our study, the cohort effect played a larger role for females than for males in VL incidence rates. We observed higher incidence and mortality relative risk in the recent birth cohort about the period 1970–71 than in the earlier birth cohorts. One of the possible explanations for the higher incidence in the recent birth cohort can be the apparent increase in the reporting rather than an actual change in levels. Moreover, the information technology revolution in recent periods has brought significant changes in the treatment-seeking which might have led to higher incidence and mortality risk in the recent cohorts. The cohort effect showed a declining pattern in the incidence relative risk among birth cohort 1900–34 and further witnessed an increase later on. This increase in the incidence and mortality rates could be attributed to the seasonality of the disease. However, birth cohort 1905–09 to 1960–64 witnessed a reduced mortality relative risk. However, birth cohorts 1945–49 experienced a higher incidence of relative risk than relative risk of mortality. It is only in the most recent cohort which has experienced a decline in both incidence and mortality rates (from 2014 onwards).

## Conclusion

To our knowledge, this study is the first study to provide insights into the country-wide epidemic trend and demographic patterns of visceral leishmaniosis. This study provides a comprehensive analysis of VL epidemiology in India, highlighting the importance of targeted interventions and sustained surveillance. This study uniquely identifies temporal trends using joinpoint and APC analysis, providing a detailed understanding of disease progression across different age groups. Furthermore, no previous studies with large, cohort-based samples, have been available for direct comparison, making our findings particularly significant. The observed age pattern in VL incidence and mortality underscore the need for age-specific preventive measures and targeted intervention strategies. Gender-specific interventions should be prioritized to address disparities in exposure, healthcare access, and treatment outcomes, ensuring a more equitable approach to VL prevention and control. Our extensive epidemiological investigation showed that from 1990 to 2019, there was a decline in the yearly incidence of VL in India. Although there are substantial differences in the epidemic characteristics over the period, further research is needed to explore the influence of environmental and socioeconomic factors on VL trends. Validation of these findings in other endemic regions would enhance their generalizability. A spatial–temporal analysis might provide a better insight into the distribution of the disease. In order to reduce the risk of transmission, efforts must be made to increase large-scale screening, pesticide spraying, health education that promotes behavioural change, and other integrated measures. Policymakers should utilize these findings to design targeted interventions and resource allocation strategies to advance VL elimination.

## Supplementary Information

Supplementary Material 1. 

## Data Availability

The datasets analyzed during the current study are available in the (Global Health Data Exchange) query tool produced by the IHME repository, [(http://ghdx.healthdata.org/gbd-results-tool].
