# Supplementary material for: Long-term trends of visceral leishmaniasis incidence and mortality in India 1990–2019: an application of joinpoint and age-period-cohort analysis
Source: BMC Infect Dis. 2025 Sep 29;25:1176. doi: 10.1186/s12879-025-10751-7 (PMC12482584; doi:10.1186/s12879-025-10751-7)
Supplement: Supplementary file 1 — Supplementary Material 1. [file 12879_2025_10751_MOESM1_ESM.docx]

**Long-term trends of visceral leishmaniasis incidence and mortality in India 1990-2019: an application of joinpoint and age-period-cohort analysis**

Appendix Table 1. Visceral Leishmaniasis incidence and mortality rates estimated coefficients for the age, period and cohort effects

| **Factors** | **Incidence (Coef.)** | | **Mortality (Coef.)** | |
| --- | --- | --- | --- | --- |
|  | **Male** | **Female** | **Male** | **Female** |
| **Age** |  |  |  |  |
| **0-4** | 1.252203 | 1.490065 | 0.7773715 | 1.232209 |
| **5-9** | 1.371951 | 1.376011 | 0.2269718 | 0.45098 |
| **10-14** | 0.6911629 | 0.42643 | -0.3188907 | -0.4025514 |
| **15-19** | 0.2163125 | -0.0761454 | -0.2411259 | -0.5325704 |
| **20-24** | -0.0603372 | -0.2892822 | -0.2212564 | -0.6285687 |
| **25-29** | 0.0518187 | -0.0856366 | 0.0232019 | -0.2907726 |
| **30-34** | 0.0748645 | 0.0104892 | 0.1544116 | -0.1069254 |
| **35-39** | -0.0331986 | -0.0140776 | 0.136347 | 0.0091795 |
| **40-44** | -0.2268081 | -0.1440323 | 0.041679 | 0.0363524 |
| **45-49** | -0.4941467 | -0.3614497 | -0.0842438 | -0.0281583 |
| **50-54** | -0.5877495 | -0.4140241 | -0.1903273 | -0.0645864 |
| **55-59** | -0.5228627 | -0.3474036 | -0.1307845 | -0.0139573 |
| **60-64** | -0.4071056 | -0.2905979 | -0.0252938 | 0.0265044 |
| **65-69** | -0.2789185 | -0.2300173 | 0.0367102 | 0.0577868 |
| **70-74** | -0.2857248 | -0.2475847 | -0.0130789 | 0.068923 |
| **75-79** | -0.2672455 | -0.2664878 | -0.0507796 | 0.0745092 |
| **80-84** | -0.2085207 | -0.2659912 | -0.0635603 | 0.0065046 |
| **85-89** | -0.1749875 | -0.2069061 | -0.0909433 | -0.0006122 |
| **90-94** | -0.1107078 | -0.0633592 | 0.0335915 | 0.105754 |
| **Period** |  |  |  |  |
| **1990-94** | 1.445022 | 1.440078 | 1.36273 | 1.394302 |
| **1995-99** | 0.3140502 | 0.3113337 | 0.2231144 | 0.1912441 |
| **2000-04** | 0.0122203 | 0.0123689 | -0.1335958 | -0.175337 |
| **2005-09** | 0.2063605 | 0.2082648 | 0.3321911 | 0.3198282 |
| **2010-14** | -0.387178 | -0.3838851 | -0.3115718 | -0.3178587 |
| **2015-19** | -1.590475 | -1.588161 | -1.472868 | -1.412179 |
| **Cohort** |  |  |  |  |
| **1900-04** | 0.037367 | -0.0610651 | 0.2709543 | 0.4015446 |
| **1905-09** | -0.2557645 | -0.1544768 | 0.221345 | 0.2951064 |
| **1910-14** | -0.3426431 | -0.2000579 | 0.1892555 | 0.2009252 |
| **1915-19** | -0.3303289 | -0.24516 | 0.0789018 | 0.0713275 |
| **1920-24** | -0.3230088 | -0.2567129 | -0.0282807 | -0.0178905 |
| **1925-29** | -0.2813305 | -0.2441485 | -0.0982647 | -0.1301861 |
| **1930-34** | -0.2582456 | -0.2713023 | -0.1153188 | -0.2006166 |
| **1935-39** | -0.2168585 | -0.2397905 | -0.1177452 | -0.1594342 |
| **1940-44** | -0.1443547 | -0.1795063 | -0.1298348 | -0.1562104 |
| **1945-49** | -0.1131495 | -0.134648 | -0.1538851 | -0.1556361 |
| **1950-54** | -0.1007508 | -0.094624 | -0.2118654 | -0.1459404 |
| **1955-59** | -0.0978951 | -0.0886782 | -0.2685549 | -0.1777044 |
| **1960-64** | -0.095384 | -0.097254 | -0.2882318 | -0.2030644 |
| **1965-69** | -0.0559219 | -0.0709377 | -0.268711 | -0.1789295 |
| **1970-74** | -0.00069 | -0.0309007 | -0.1789735 | -0.1308904 |
| **1975-79** | 0.0425079 | 0.013897 | -0.1229891 | -0.0695178 |
| **1980-84** | 0.0827891 | 0.0639001 | -0.117888 | -0.0258305 |
| **1985-89** | 0.1389472 | 0.1179367 | -0.0458898 | 0.0511111 |
| **1990-94** | 0.2080787 | 0.1865778 | 0.1687383 | 0.1583545 |
| **1995-99** | 0.2843474 | 0.2594325 | 0.1966845 | 0.1673293 |
| **2000-04** | 0.3899291 | 0.3671951 | 0.2436849 | 0.1355724 |
| **2005-09** | 0.4675047 | 0.4459641 | 0.2431101 | 0.1276839 |
| **2010-14** | 0.4919843 | 0.4680532 | 0.2573804 | 0.1307364 |
| **2015-19** | 0.4728705 | 0.4463063 | 0.2763781 | 0.01216 |
